# Mucosal Injuries due to Ribosome-Inactivating Stress and the Compensatory Responses of the Intestinal Epithelial Barrier

**DOI:** 10.3390/toxins3101263

**Published:** 2011-10-20

**Authors:** Yuseok Moon

**Affiliations:** 1 Laboratory of Systems Mucosal Biomodulation, Department of Microbiology and Immunology, Medical Research Institute, Pusan National University School of Medicine, Yangsan 626-870, Korea; Email: moon@pnu.edu; Tel.: +82-51-510-8094; Fax: +82-55-382-8090

**Keywords:** ribotoxic stress, intestinal epithelial barrier, mucosal toxicity

## Abstract

Ribosome-inactivating (ribotoxic) xenobiotics are capable of using cleavage and modification to damage 28S ribosomal RNA, which leads to translational arrest. The blockage of global protein synthesis predisposes rapidly dividing tissues, including gut epithelia, to damage from various pathogenic processes, including epithelial inflammation and carcinogenesis. In particular, mucosal exposure to ribotoxic stress triggers integrated processes that are important for barrier regulation and re-constitution to maintain gut homeostasis. In the present study, various experimental models of the mucosal barrier were evaluated for their response to acute and chronic exposure to ribotoxic agents. Specifically, this review focuses on the regulation of epithelial junctions, epithelial transporting systems, epithelial cytotoxicity, and compensatory responses to mucosal insults. The primary aim is to characterize the mechanisms associated with the intestinal epithelial responses induced by ribotoxic stress and to discuss the implications of ribotoxic stressors as chemical modulators of mucosa-associated diseases such as ulcerative colitis and epithelial cancers.

## 1. Introduction

The mucosal epithelium constitutes a physical and functional barrier between the host and components of the external environment, including nutrients, microbes, and toxicants [1,2]. In particular, the intestinal lining consists of an epithelial monolayer in which epithelial cells associate with one another at the apical junctional complex via junctional structures. The junctional complex limit the paracellular passage of fluids and solutes and maintain physical integrity as a barrier to microbes and xenobiotics while permeable to nutrients and essential biomaterials important for homeostasis and growth [3,4]. In addition to the physical functions of the epithelial barrier, gut epithelial cells also interact and communicate with enteric bacteria and other immune-related host cells such as leukocytes [5,6]. Various dysfunctions of the epithelial barrier have been identified in human intestinal mucosal diseases, including inflammatory bowel disease (IBD) and epithelial cancers [2,7,8]. Increased paracellular permeability has been observed in the epithelial lining of both acutely inflamed and chronically damaged intestines. In addition to the upper epithelial barrier, the lower barrier of the gut lining regulates antigen traffic via intensive crosstalk between gut epithelial cells and underlying leukocytes. During the disease process, luminal antigens, including commensal bacteria, can pass through the loose upper barrier and directly interact with underlying immune-related cells, causing severe immune hypersensitivity. Therefore, a better understanding of barrier alteration in human intestinal disorders may provide insights into emerging treatment therapies for these pathologies.

Ribosome-inactivating (ribotoxic) xenobiotics belong to a large family of ribonucleolytic agents. A number of ribosome-inactivating proteins (RIPs) can irreversibly cleave 28S ribosomal RNA at a single phosphodiester bond within a universally conserved sequence known as the sarcin-ricin loop, and this cleavage leads to the dysfunction of peptidyltransferase and subsequent global translational arrest [9,10,11,12]. RIPs have been identified in several biological kingdoms, including plants (e.g., ricin, abrin, agrostin, saporin, and ebulin), fungi (e.g., α-sarcin and restrictocin), and bacteria (e.g., shiga toxin). Ribosomal cleavage can also occur with non-protein ribotoxic stress triggered by physical or chemical insults, including ultraviolet (UV) irradiation, trichothecene mycotoxins, palytoxin, and anisomycin antibiotics, which also interfere with peptidyltransferase activity by directly or indirectly modifying 28S rRNA although different cellular parts can be previously reported as their major targets of some ribotoxic insults [13,14]. In addition to ribosomal cleavage, ribotoxic xenobiotics can phosphorylate serine 51 on the alpha subunit of eukaryotic translation initiation factor 2 (eIF2α), which leads to global translational arrest [15]. Therefore, tissues with high rates of division, such as lymphoid tissue and mucosal epithelium, are more susceptible to ribotoxic insults than tissues with slower division rates [16,17,18]. Epidemiological studies have led to the identification of several potential links between ribotoxic intoxication and human mucosal epithelial illnesses, ranging from acute mucosal inflammatory disease to chronic illness, including epithelial malignancy [19,20,21]. Ribotoxic xenobiotics alter intestinal integrity, and this leads to blunting of intestinal villi, swelling of interepithelial spaces, mucous secretion, dysregulation of nutrient absorption, and mucosal immune hyper-activation, all of which are associated with diarrhea, weight loss, and ulcerative colitis (UC) in experimental animals [22,23,24,25,26]. Moreover, it has been suggested that chronic exposure to some types of ribotoxic stress in animals and human can lead to epithelial tumor development [27,28,29,30]. 

The present review describes mechanistic insights associated with the modulation of intestinal epithelial integrity by ribotoxic stress during human intestinal epithelial pathogenesis. The purpose of this review is to provide a deep understanding of the key roles played by intestinal epithelial cells within the epithelial barrier system in both healthy subjects and those exposed to ribotoxic stress. In particular, this review focuses on the modulation of epithelial junction regulation, epithelial transporting system, epithelial cytotoxicity, and compensatory responses to the mucosal insults associated with IBD and epithelial cancers. 

## 2. Ribotoxic Stress-Mediated Barrier Disruption

### 2.1. Modulation of the Epithelial Transport System

After exposure to mucosal ribotoxic stress, gut epithelial cells use defensive transporters to excrete the toxins in the feces. The most well known is the ATP-binding cassette (ABC) transporter, which is specialized for exporting toxic xenobiotics. Intestinal epithelial cells also use some ABC transporters, such as the multidrug resistance-associated protein 1 (MDR1, ABCB1) and MRP2 (ABCC2), to export ribotoxic trichothecenes [31,32]. MDR1 is expressed highly in the intestinal epithelia, renal tubular epithelia, canalicular membrane of hepatocytes, cerebral capillary endothelial cells, testes, and ovaries. MDR1 exports a broad range of hydrophobic compounds, and thus functions as a biological barrier by preventing the exposure of tissues to toxic substances. MDR1 is also a secretory transporter that limits the entry of toxic xenobiotics into the gut epithelial cells. In contrast to the defensive roles played by ABC transporters, retroviral induction of human *MDR1* cDNA, which encodes ABCB1, triggers major accumulation of globotriaosylceramide, a glycosphingolipid receptor for the ribotoxic shiga toxin, leading to a million-fold increase in barrier cell sensitivity to toxins [33]. The human *MDR1* gene is located on chromosome 7 (7q21.1), a susceptibility locus for inflammatory bowel disease (IBD) [34,35]. A meta-analysis study demonstrated that a significant number of IBD patients harbor mutations in this gene and develop a severe spontaneous colitis that is characterized by impaired functionality of the intestinal barrier and immune reactivity to luminal xenobiotics, such as ribotoxic agents. Moreover, ribotoxic xenobiotics modulate nutrient transport by interfering with the d-glucose/d-galactose sodium-dependent transporter (SGLT1), the d-fructose transporter GLUT5, active/passive l-serine transporters, and the passive transporters of d-glucose (GLUT) [23,36]. The selective effects of ribtoxic stress on intestinal nutrient transport may account for the malnutrition and weight loss observed in toxin-exposed animals and human. A suppressed nutrient transport system provides a potential mechanism by which ribotoxic stress could induce diarrhea in animals and humans. For instance, exposure to low amounts of ribotoxic deoxynivalenol can cause aqueous diarrhea by inhibiting the intestinal SGLT1 transporter, which results in an imbalance of epithelial cellular water due to decreased d-glucose-associated water absorption. Moreover, exposure to toxic levels of deoxynivalenol may inhibit SGLT1 function and disrupt the epithelial barrier, leading to inflammatory diarrhea.

### 2.2. Disrupted Epithelial Junctions due to Ribotoxic Stress

The intestinal mucosal barrier has evolved to maintain a delicate balance between the absorption of essential nutrients and the prevention of harmful xenobiotic entry and responses. Junction structural alterations can be detected in the gut epithelial barrier in both human IBD and experimental models of intestinal inflammation. Leaky gut symptoms are linked to an increase in intestinal permeability and a decrease in transepithelial resistance. The intestinal epithelial junctions consist of desmosomes, adherent junctions, and tight junctions, all of which modulate both intercellular adhesion and paracellular transport. E-cadherin plays an important role in maintaining the integrity of the intestinal barrier; its cellular localization is disrupted in patients with Crohn’s disease (CD) [37]. However, the abnormal expression and dislocalization of tight junction structural proteins, such as hyperphosphorylated occludins, zonula occludens, and claudin family proteins, are frequently observed in IBD pathogenesis [38]. The tight junctions, located at the apical end of the intercellular space, are regulated highly by cytokines, which play critical roles in the modulation of intestinal barrier function. Pro-inflammatory cytokines, such as tumor necrosis factor (TNF)-α, interferon γ, interleukin (IL)-1β, IL-13, and a cellular ligand for herpes virus entry mediator and lymphotoxin receptor (LIGHT), promote barrier dysfunction by inhibiting the transcription of junction proteins and inducing cytoskeleton-mediated redistribution of tight junction proteins [39,40]. For instance, some pro-inflammatory cytokines enhance the expression of myosin light-chain kinase, which leads to myosin II re-arrangement to allow interaction with tight junction proteins. Re-organized tight junction proteins are then able to exit the complex via the endocytic pathway. In contrast, regulatory cytokines, including transforming growth factor (TGF)-β, are involved in the re-constitution of tight junctional complexes [41]. The gut epithelia in IBD patients displays a leaky barrier of decreased junctional complexity, with a lower number of tight junction strands, reduced depth of the primary tight junctional meshwork, increased apoptotic rate, and appearance of strand discontinuity, all of which have been associated closely with increased uptake of luminal food and bacterial antigens [42]. Because epithelial barrier disruption increases host susceptibility to acute microbial infections and to chronic hypersensitive diseases, including IBD, various ribotoxic stresses have been studied extensively for their effects on the tight junctions of the epithelial barrier [43,44,45,46,47,48,49]. In response to ribotoxic deoxynivalenol, the intestinal epithelial barrier in the pig (the most susceptible species) demonstrated injury by decreased expression of the tight junction protein, claudin-4 [47,48]. Decreased claudin-4 leads to a loss of epithelial cell integrity and disruption of epithelial polarity. A recent study demonstrated that, depending on the route of toxin exposure (direct apical versus basolateral via the circulation), the polarized gut epithelial cell layer responded differently to ribotoxic deoxynivalenol. Basolateral exposure of the gut barrier causes significantly more vulnerability to the tight junctional breakdown than exposure to the apical surface [44]. The reduced quantity of tight junction proteins is mediated by ribotoxin-activated p44/42 extracellular signal-regulated kinase (ERK) MAPK. Ribotoxic stress induces various pro-inflammatory cytokines, including TNF-α and IL-1β. These cytokines may play important roles in dysregulating the tight junctional complex and decreasing epithelial integrity, as suggested in IBD pathogenesis. The specific association between pro-inflammatory cytokines and epithelial barrier disruption is implicated in ribotoxin ricin-intoxicated animal model [26]. Taken together, ribotoxic stresses promote the leaky gut environment by disrupting junctional structures, which allows for the translocation of luminal bacteria and for the subsequent over-stimulation of underlying lymphoid systems. The ribotoxic stress may facilitate overload of luminal contents (e.g., enteric bacteria) through the leaky barrier, which may demonstrate the potent etiological factors of human IBD. 

## 3. Mucosal Cell Death due to Ribotoxic Stress and Epithelial Counteractions

### 3.1. MAPK-Linked Epithelial Cell Death and Reconstitution in Response to Ribotoxin Intoxication

Epithelia with regions of apoptosis are susceptible to conductive leakage, which leads to barrier dysfunction, bacterial translocation and subsequent infection. Increased epithelial apoptosis is another critical factor of compromised barrier integrity in both UC and CD patients [50,51,52,53]. In early stages of mild to moderate inflammation in UC, apoptosis accounts for approximately half of the impaired colorectal conductivity whereas the other half is caused by degradation of tight junctions in non-apoptotic areas [54]. Erosion and ulcer-type lesions associated with the severe mucosal and systemic inflammatory response dominate the late stages of the disease [42]. In dextran sulfate sodium (DSS)-induced colitis, the colonic epithelia demonstrate increased apoptosis and decreased proliferation, which may lead to the breakdown of the epithelial barrier, and thus facilitate the mucosal invasion of enteric bacteria [55]. During IBD pathogenesis, some species of commensal enteric bacteria, including *E. coli* O4, may breach the epithelial barrier by forming apoptotic loci or focal leaks at points of bacterial penetration. Mucosal ribotoxic xenobiotics can also induce epithelial apoptosis in the gastrointestinal tract in different exposure models [18,44,45,46,47,48,49,50,51,52,53,54,55,56]. Intestinal epithelia are more resistant than lymphocytes to the cytotoxic actions associated with ribotoxic stress, but the local concentration of ribotoxins in the gastrointestinal tract following dietary ingestion is generally much higher than ribotoxin levels in the circulation and other distributed tissues. Thus, damage-mediated action of a higher intensity could be expected in the intestinal epithelium. However, intramuscular exposure to ribotoxic stress can lead to severe small intestinal injuries as well as the infiltration of large numbers of plasma cells into the lamina propria and subsequent apoptosis of mucosal lymphocytes [46,56]. Regarding signaling pathways, ribotoxic agents activate p38 MAPK and Jun *N*-terminal kinase (JNK), which are involved in pro-apoptotic caspase 3 activation in intestinal epithelia [18,57]. MAPK also can mediate negative regulation of apoptosis induction; e.g., extracellular signal-regulated kinases (ERK) 1/2 can directly phosphorylate caspase 9 in response to growth factor [58]. Caspase 9 phosphorylation is critical to restraining apoptosis during mitotic arrest by Bcl2 phosphorylation, whereas the proteolytic cascade of caspase 9 leads to apoptosis. The ERK1/2 signal also can protect cells from ulcerative insults by enhancing epithelial proliferation and cell survival [59]. ERK1/2 signal is activated in response to ribotoxic stress-mediated pro-apoptotic signals to mediate expression of early growth response gene 1 product (EGR1) [60], which can trigger epithelial reconstitution as well as mitotic cytokine production. Ultimately, the fate of ribotoxin-exposed epithelial cells is determined by the counteracting balance between pro-apoptotic signals and survival responses, dependant on the signaling context. 

### 3.2. Other Ribotoxic Mediators of Epithelial Apoptosis

Some ribotoxic agent-induced cell deaths are independent of MAPK activation. Several trichothecenes, including acetyl T-2, T-2 toxin, and verrucarin, inhibit protein synthesis without activating JNK and lead to caspase activation, which indicates that JNK and p38 kinases are not necessary for caspase activation [57]. As another MAPK-independent pathway, ribotoxic stress can elicit endoplasmic reticulum (ER) stress. ER stress induces calcium (Ca^2+^) release in leukocytes—Ca^2+^-dependent calpain activation and subsequent pro-apoptotic caspase 8 activation [61]. Ribotoxic trichothecenes can also induce ER stress in gut epithelial cells, but it is not linked to cellular apoptosis [62]. Ribotoxin-induced epithelial ER stress mediates pro-inflammatory cytokine production instead of apoptosis [63], which may contribute to the disruption of epithelial tight junctions, as suggested in section 2.2. However, since local intestinal ribotoxic doses are generally higher than those in circulation, extremely high doses of ribotoxic agents can induce epithelial cell death. In addition to caspase-mediated cell death, ribotoxic anisomycin induces macrophage inhibitory cytokine 1 (MIC-1) gene expression, which is a critical inducer of intestinal cellular apoptosis and barrier disruption [64]. MIC-1, which is also known as PTGF-β, PLAB, GDF15, PDF, NAG-1, and PL74, is a cytokine from the transforming growth factor-β superfamily and is involved in epithelial pathogenesis [65,66,67]. Little to no detectable expression of MIC-1 is observed in normal epithelial cells. However, the level of MIC-1 expression rises dramatically with epithelial neoplastic transformation, and MIC-1 expression increases further in response to a variety of anti-tumorigenic stimuli, including gamma irradiation and chemo-preventive agents—the latter includes non-steroidal anti-inflammatory drugs (NSAIDs) and natural products [68,69]. NSAIDs induce ulcerative lesions similar to those induced by ribotoxic xenobiotics, and the 		pro-apoptotic action of NSAIDs in the gut epithelium is mediated by MIC-1 protein though ER stress [70]. Although the mechanism of MIC-1-mediated apoptosis is not clearly understood in the gut epithelium, several lines of evidence indicate that since the promoter of MIC-1 has a potential promoter binding site for p53 protein, MIC-1 can mediate p53-dependent growth suppression [68,71,72]. However, MIC-1 can induce apoptosis independent of the p53 pathway [73]. MIC-1 is expressed in colorectal cancer tissues and has strong associations with serum levels and malignancy in the gut mucosa [65]. Moreover, MIC-1 induces tumor metastasis by triggering the FAK-RhoA signaling pathway, which leads to actin reorganization [74]. Chronic exposure to the ribotoxic stress can maintain prolonged MIC-1 expression, which may be linked to intestinal neoplastic progression. Therefore, further investigations are needed to examine other MIC-1-mediated effects on epithelial cells chronically exposed to ribotoxic agents, whereas MIC-1 effects induced by acute exposure can be pro-apoptotic.

## 4. Compensatory Responses of Epithelial Cells to Mucosal Ulceration

In the DSS-induced UC model, DSS binding is involved in the cell cycle arrest of intestinal monolayer cells [55,75]. DSS directly arrests cell cycles in the Caco-2 epithelial monolayer, but cell proliferation increases in the chronically exposed gut epithelia [76], a finding supported by clinical studies [77,78]. Therefore, epithelial cell growth promotion in DSS-induced colitis and UC patients may be a compensatory repair process for colitis-induced erosion. However, the accelerated epithelial cell turnover due to epithelial injuries and mitogenic pro-inflammatory stimulation may predispose gut epithelia to DNA damage. Thus, increased proliferation of gut epithelial cells in UC patients can be associated with dysplasia in colorectal neoplasia. In contrast with the epithelial injuries by pro-apoptotic MIC-1, MIC-1 transcriptional modulator, activating transcription factor 3 (ATF3), plays a protective role against mucosal ribotoxic stress [79]. ATF3 is a transcription factor of the ATF/cyclic AMP response element-binding family and contains a basic region/leucine zipper DNA-binding motif that binds to the cyclic AMP response element consensus sequence [80]. ATF3 often is induced by external stress signals like ischemic injuries, mutagens, carcinogens, mitogenic cytokines, and endoplasmic reticulum (ER) stresses in addition to ribotoxic stress [81,82]. The counterbalance of ATF3 and ribotoxic stress-induced apoptosis to maintain epithelial homeostasis in response to the ribotoxic stresses may be a critical point of mucosal regulation. In particular, erosion of the epithelial barrier can trigger compensatory epithelial proliferation mediated by ATF3 protein. 

Cell cycle arrest after stress may provide the time necessary to re-establish cellular homeostasis, particularly in response to cytotoxicity [83,84]. Some toxicological or physical triggering by ribotoxic stresses stimulates p53- or p21-dependent cell cycle arrest and apoptosis [85,86]. Moreover, human intestinal epithelial cells undergo G_2_/M phase arrest in response to mild mucosal ribotoxic stress without a significant increase in apoptotic cell death. According to a SCOOP study, plausibly realistic intestinal concentrations (160-2000 ng/mL) of ribotoxic deoxynivalenol do not induce intestinal epithelial apoptosis [24]. Gene expression of p21 can be induced by ribotoxic deoxynivalenol treatment with no increase in p53 protein levels, suggesting p53-independent p21 induction [87]. In terms of transcriptional regulation, p21 is the transcriptional target of p53 by DNA damaging agents [88,89]. The active ulcerative region exhibits increased apoptosis and cell cycle arrest via enhanced levels of p53 and p21 protein during the early stage of human IBD and DSS-induced colitis [77]. However, p21 is down-regulated as the disease becomes chronic and UC-related neoplasm develops. p21 can also mediate mucosal pro-inflammatory cytokine production and NF-κB-dependent signaling through p21-dependent kinase (PAK) [90,91]. In addition to the p21-mediated epithelial cell cycle arrest, p21 can be linked to ribotoxic stress-mediated pro-inflammatory stimulation in the gut epithelia. Taken together, ribotoxic stress may affect dynamic expression of p21 protein in gut epithelial pathogenesis, in beneficial or harmful ways depending on the disease stages. 

## 5. Link of Chronic Mitogenic Stimulation to Tumor Promotion by Chronic Ribotoxic Exposure

In terms of cancer therapy, investigations have focused on acute use of the ribotoxic agents as anti-tumor agents [92,93,94]. Particular interests in ricin and related ribotoxic proteins were stimulated by the higher toxicity to cancerous cells than non-cancerous cells [95]. However, evidence of the involvement of chronic ribotoxic stress in epithelial tumor promotion have been suggested in several experimental and epidemiological studies [27,28,29,30]. A local Chinese population consuming a diet with high ribotoxic trichothecenes, including nivalenol and diacetoxyscirpenol, had a higher risk of developing esophageal cancer than a group exposed to less or negligible amounts of toxins. Animal experiments also support this by demonstrating that the intermittent epidermal applications of nivalenol, alternated with 12-tetradeconoyl-phorbol-13-acetate (TPA) application enhance the risk of papillomas and carcinomas [29]. The most well-investigated ribotoxic tumor promoters are palytoxin and UV irradiation, which are also strong cellular mitogens in injured epithelial tissue [30,96]. Mechanistically, common downstream targets of the ribotoxic tumor promoters are MAPK-activated proliferative signals. Palytoxin binds to the Na^+^, K^+^-ATPase, which triggers ERK MAPK during tumor promotion. UV irradiation can activate stress-activated kinase (SAPK) and epidermal growth factor receptor (EGFR)-linked protein kinase B signals in an alternative manner to decrease ERK1/2 MAPK kinases in normal human keratinocytes. Although the acute ribotoxic UV can lead to cell cycle arrest by inactivating some MAPK signals such as ERK1/2, alternative mitogenic signals via EGFR can thereafter support proliferation of the surviving cells. Chronic intermittent mitogenic insults can cause gut epithelial tissue injuries, but subsequent surviving epithelial cells or epithelial stem cells would be provided with growth advantage via alternative signals, which can be linked to epithelial tumor promotion in response to ribotoxic stress in the gut. 

## 6. Conclusion

**Figure 1:**
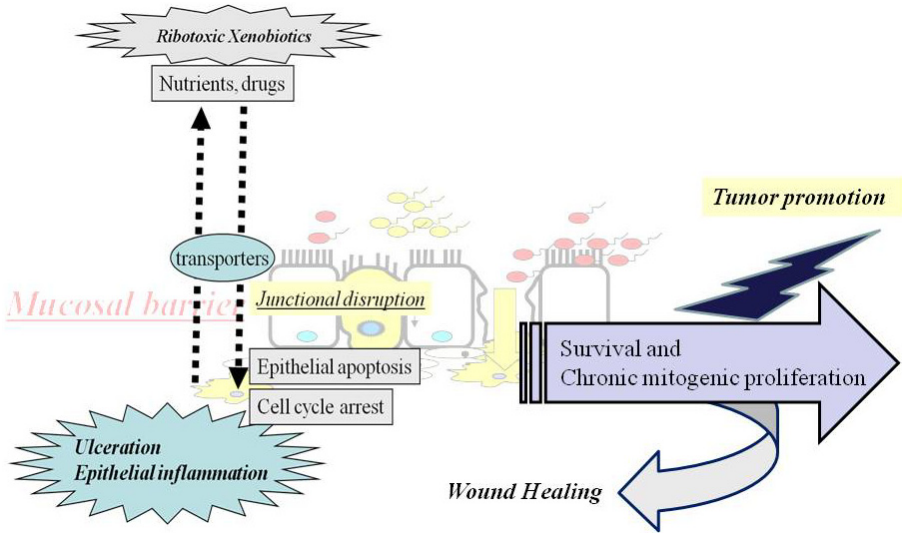
A putative scheme for epithelial responses to muco-active ribotoxic stressors and subsequent pathologic fates after chronic exposure.

Different modulations of intestinal epithelial integrity by ribotoxic xenobiotics have been suggested in terms of human intestinal epithelial diseases, including ulcerative colitis and epithelial tumors (Figure 1). (1) Primarily, the epithelial barrier is important for mucosal defense and homeostasis. After exposure to mucosal ribotoxic xenobiotics, gut epithelia employ defensive transporters to excrete the toxins. Excretion or absorption of ribotoxins can be modulated via ABC transporters, including MDR1 and MRP2. Moreover, ribotoxic xenobiotics also modulate nutrient transporting by interfering with various nutrient transporter systems, which might account for the malnutrition and weight loss in exposed mammals. (2) In addition to the xenobiotic transporting system, the epithelial junction is also critical for maintaining epithelial barrier integrity. Generally, IBD patients with abnormal expression and dislocalization of structural proteins of tight junction are susceptible to mucosal insults. Ribotoxic stresses promote a leaky gut environment by disrupting the junctional structures, which can facilitate subsequent discharging of luminal contents and mucosal immune dysregulation as implicated in human IBD. (3) Increased epithelial apoptosis is another etiological factor of broken barrier integrity in both UC and CD patients. Locally intensified ribotoxins cause injuries to the gut mucosal epithelia in MAPK-dependent ways or via other pro-apoptotic pathways. (4) In response to the cytotoxic effects of mucosal insults, gut epithelia also can trigger cell-arresting or survival responses. Cell cycle arrest after mucosal ribotoxic stress may provide the time necessary to re-establish cellular homeostasis. Some ribotoxic stress may affect the dynamic expression of cell cycle-modulators such as p21 protein in gut epithelial pathogenesis, in beneficial or harmful ways, depending on the disease stage. The epithelial cell growth promotion in IBD patients might be a compensatory repair process from colitis-induced erosion. Survival mitogenic signals are needed for epithelial proliferation and to compensate for the eroded barrier. However, prolonged epithelial cell turnover and mitogenic pro-inflammatory stimulation predispose the gut epithelia to undergo genetic transformation and a growth advantage over the surrounding cells, which can thus be associated with dysplasia in intestinal neoplasia. The chronic exposure to ribotoxic stress can be linked to epithelial tumor promotion, as suggested in several experimental and epidemiological studies. One common downstream target of the ribotoxic tumor promoters are MAPK-activated proliferative signals. In future studies, the mechanistically suggested mediators described in the present review need to be systematically assessed in the human body and in specimens from the normal and clinically diseased groups. More translation approaches should be made for better treatment and prevention of environmentally linked human epithelial diseases.

## References

[1] Catalioto R.M., Maggi C.A., Giuliani S. (2011). Intestinal epithelial barrier dysfunction in disease and possible therapeutical interventions.. Curr. Med. Chem..

[2] Laukoetter M.G., Nava P., Nusrat A. (2008). Role of the intestinal barrier in inflammatory bowel disease.. World J. Gastroenterol..

[3] Amasheh S., Fromm M., Gunzel D. (2011). Claudins of intestine and nephron-A correlation of molecular tight junction structure and barrier function. Acta Physiol. (Oxf.).

[4] Shimizu M. (2010). Interaction between food substances and the intestinal epithelium.. Biosci. Biotechnol. Biochem..

[5] Hofman P.M. (2010). Pathobiology of the neutrophil-intestinal epithelial cell interaction: Role in carcinogenesis.. World J. Gastroenterol..

[6] Kunisawa J., Kiyono H. (2010). Aberrant interaction of the gut immune system with environmental factors in the development of food allergies.. Curr. Allergy Asthma Rep..

[7] Medema J.P., Vermeulen L. (2011). Microenvironmental regulation of stem cells in intestinal homeostasis and cancer.. Nature.

[8] Roda G., Sartini A., Zambon E., Calafiore A., Marocchi M., Caponi A., Belluzzi A., Roda E. (2010). Intestinal epithelial cells in inflammatory bowel diseases.. World J. Gastroenterol..

[9] Girbes T., Ferreras J.M., Arias F.J., Stirpe F. (2004). Description, distribution, activity and phylogenetic relationship of ribosome-inactivating proteins in plants, fungi and bacter. Mini. Rev. Med. Chem..

[10] Lacadena J., Alvarez-Garcia E., Carreras-Sangra N., Herrero-Galan E., Alegre-Cebollada J., Garcia-Ortega L., Onaderra M., Gavilanes J.G., Martinez del Pozo A. (2007). Fungal ribotoxins: Molecular dissection of a family of natural killers.. FEMS Microbiol. Rev..

[11] Ng T.B., Wong J.H., Wang H. (2010). Recent progress in research on ribosome inactivating proteins.. Curr. Protein Pept. Sci..

[12] Stirpe F., Battelli M.G. (2006). Ribosome-inactivating proteins: Progress and problems.. Cell Mol. Life Sci..

[13] Iordanov M.S., Pribnow D., Magun J.L., Dinh T.H., Pearson J.A., Magun B.E. (1998). Ultraviolet radiation triggers the ribotoxic stress response in mammalian cells.. J. Biol. Chem..

[14] Li M., Pestka J.J. (2008). Comparative induction of 28S ribosomal RNA cleavage by ricin and the trichothecenes deoxynivalenol and T-2 toxin in the macrophage.. Toxicol. Sci..

[15] Rzymski T., Harris A.L. (2007). The unfolded protein response and integrated stress response to anoxia.. Clin. Cancer Res..

[16] Bunyard P., Handley M., Pollara G., Rutault K., Wood I., Chaudry M., Alderman C., Foreman J., Katz D.R., Chain B.M. (2003). Ribotoxic stress activates p38 and JNK kinases and modulates the antigen-presenting activity of dendritic cells.. Mol. Immunol..

[17] Instanes C., Hetland G. (2004). Deoxynivalenol (DON) is toxic to human colonic, lung and monocytic cell lines, but does not increase the IgE response in a mouse model for allergy.. Toxicology.

[18] Smith W.E., Kane A.V., Campbell S.T., Acheson D.W., Cochran B.H., Thorpe C.M. (2003). Shiga toxin 1 triggers a ribotoxic stress response leading to p38 and JNK activation and induction of apoptosis in intestinal epithelial cells.. Infect. Immun..

[19] Luo Y., Yoshizawa T., Katayama T. (1990). Comparative study on the natural occurrence of Fusarium mycotoxins (trichothecenes and zearalenone) in corn and wheat from high- and low-risk areas for human esophageal cancer in China.. Appl. Environ. Microbiol..

[20] Li F.Q., Li Y.W., Luo X.Y., Yoshizawa T. (2002). Fusarium toxins in wheat from an area in Henan Province, PR China, with a previous human red mould intoxication episode.. Food Addit. Contam..

[21] Bhat R.V., Beedu S.R., Ramakrishna Y., Munshi K.L. (1989). Outbreak of trichothecene mycotoxicosis associated with consumption of mould-damaged wheat production in Kashmir Valley, India. Lancet.

[22] Bouhet S., Oswald I.P. (2005). The effects of mycotoxins, fungal food contaminants, on the intestinal epithelial cell-derived innate immune response.. Vet. Immunol. Immunopathol..

[23] Maresca M., Mahfoud R., Garmy N., Fantini J. (2002). The mycotoxin deoxynivalenol affects nutrient absorption in human intestinal epithelial cells.. J. Nutr..

[24] Sergent T., Parys M., Garsou S., Pussemier L., Schneider Y.J., Larondelle Y. (2006). Deoxynivalenol transport across human intestinal Caco-2 cells and its effects on cellular metabolism at realistic intestinal concentrations.. Toxicol. Lett..

[25] Stearns-Kurosawa D.J., Collins V., Freeman S., Tesh V.L., Kurosawa S. (2010). Distinct physiologic and inflammatory responses elicited in baboons after challenge with Shiga toxin type 1 or 2 from enterohemorrhagic *Escherichia coli*.. Infect. Immun..

[26] Yoder J.M., Aslam R.U., Mantis N.J. (2007). Evidence for widespread epithelial damage and coincident production of monocyte chemotactic protein 1 in a murine model of intestinal ricin intoxication.. Infect. Immun..

[27] Craddock V.M., Hill R.J., Henderson A.R. (1988). Acute and chronic effects of diacetoxyscirpenol on cell replication in rat esophagus and stomach.. Cancer Lett..

[28] Hsia C.C., Wu J.L., Lu X.Q., Li Y.S. (1988). Natural occurrence and clastogenic effects of nivalenol, deoxynivalenol, 3-acetyl-deoxynivalenol, 15-acetyl-deoxynivalenol, and zearalenone in corn from a high-risk area of esophageal cancer.. Cancer Detect. Prev..

[29] Hsia C.C., Wu Z.Y., Li Y.S., Zhang F., Sun Z.T. (2004). Nivalenol, a main Fusarium toxin in dietary foods from high-risk areas of cancer of esophagus and gastric cardia in China, induced benign and malignant tumors in mice.. Oncol. Rep..

[30] Wattenberg E.V. (2007). Palytoxin: Exploiting a novel skin tumor promoter to explore signal transduction and carcinogenesis.. Am. J. Physiol. Cell Physiol..

[31] Tep J., Videmann B., Mazallon M., Balleydier S., Cavret S., Lecoeur S. (2007). Transepithelial transport of fusariotoxin nivalenol: Mediation of secretion by ABC transporters.. Toxicol. Lett..

[32] Videmann B., Tep J., Cavret S., Lecoeur S. (2007). Epithelial transport of deoxynivalenol: Involvement of human P-glycoprotein (ABCB1) and multidrug resistance-associated protein 2 (ABCC2).. Food Chem. Toxicol..

[33] Lala P., Ito S., Lingwood C.A. (2000). Retroviral transfection of Madin-Darby canine kidney cells with human MDR1 results in a major increase in globotriaosylceramide and 10(5)- to 10(6)-fold increased cell sensitivity to verocytotoxin. Role of p-glycoprotein in glycolipid synthesis. J. Biol. Chem..

[34] Ho G.T., Soranzo N., Nimmo E.R., Tenesa A., Goldstein D.B., Satsangi J. (2006). ABCB1/MDR1 gene determines susceptibility and phenotype in ulcerative colitis: Discrimination of critical variants using a gene-wide haplotype tagging approach.. Hum. Mol. Genet..

[35] Staley E.M., Schoeb T.R., Lorenz R.G. (2009). Differential susceptibility of P-glycoprotein deficient mice to colitis induction by environmental insults.. Inflamm. Bowel Dis..

[36] Awad W.A., Aschenbach J.R., Setyabudi F.M., Razzazi-Fazeli E., Bohm J., Zentek J. (2007). In vitro effects of deoxynivalenol on small intestinal D-glucose uptake and absorption of deoxynivalenol across the isolated jejunal epithelium of laying hens.. Poult. Sci..

[37] Muise A.M., Walters T.D., Glowacka W.K., Griffiths A.M., Ngan B.Y., Lan H., Xu W., Silverberg M.S., Rotin D. (2009). Polymorphisms in E-cadherin (CDH1) result in a mis-localised cytoplasmic protein that is associated with Crohn’s disease.. Gut.

[38] Edelblum K.L., Turner J.R. (2009). The tight junction in inflammatory disease: Communication breakdown.. Curr. Opin. Pharmacol..

[39] Schwarz B.T., Wang F., Shen L., Clayburgh D.R., Su L., Wang Y., Fu Y.X., Turner J.R. (2007). LIGHT signals directly to intestinal epithelia to cause barrier dysfunction via cytoskeletal and endocytic mechanisms.. Gastroenterology.

[40] Suenaert P., Bulteel V., Lemmens L., Noman M., Geypens B., Van Assche G., Geboes K., Ceuppens J.L., Rutgeerts P. (2002). Anti-tumor necrosis factor treatment restores the gut barrier in Crohn’s disease.. Am. J. Gastroenterol..

[41] Planchon S., Fiocchi C., Takafuji V., Roche J.K. (1999). Transforming growth factor-beta1 preserves epithelial barrier function: Identification of receptors, biochemical intermediates, and cytokine antagonist. J. Cell Physiol..

[42] Schulzke J.D., Ploeger S., Amasheh M., Fromm A., Zeissig S., Troeger H., Richter J., Bojarski C., Schumann M., Fromm M. (2009). Epithelial tight junctions in intestinal inflammation.. Ann. N. Y. Acad. Sci..

[43] De Walle J.V., Sergent T., Piront N., Toussaint O., Schneider Y.J., Larondelle Y. (2010). Deoxynivalenol affects *in vitro* intestinal epithelial cell barrier integrity through inhibition of protein synthesis.. Toxicol. Appl. Pharmacol..

[44] Diesing A.K., Nossol C., Danicke S., Walk N., Post A., Kahlert S., Rothkotter H.J., Kluess J.  (2011). Vulnerability of polarised intestinal porcine epithelial cells to mycotoxin deoxynivalenol depends on the route of application. PLoS One.

[45] Diesing A.K., Nossol C., Panther P., Walk N., Post A., Kluess J., Kreutzmann P., Danicke S., Rothkotter H.J., Kahlert S. (2011). Mycotoxin deoxynivalenol (DON) mediates biphasic cellular response in intestinal porcine epithelial cell lines IPEC-1 and IPEC-J2.. Toxicol. Lett..

[46] Liu L., Gao H., Li J., Dong Y., Liu N., Wan J., Liu W., Sun Y., Xu M. (2009). Analysis of intestinal injuries induced by ricin *in vitro* using SPR technology and MS identification.. Int. J. Mol. Sci..

[47] Pinton P., Braicu C., Nougayrede J.P., Laffitte J., Taranu I., Oswald I.P. (2010). Deoxynivalenol impairs porcine intestinal barrier function and decreases the protein expression of claudin-4 through a mitogen-activated protein kinase-dependent mechanism.. J. Nutr..

[48] Pinton P., Nougayrede J.P., Del Rio J.C., Moreno C., Marin D.E., Ferrier L., Bracarense A.P., Kolf-Clauw M., Oswald I.P. (2009). The food contaminant deoxynivalenol, decreases intestinal barrier permeability and reduces claudin expression. Toxicol. Appl. Pharmacol..

[49] Van de Walle J., During A., Piront N., Toussaint O., Schneider Y.J., Larondelle Y. (2010). Physio-pathological parameters affect the activation of inflammatory pathways by deoxynivalenol in Caco-2 cells.. Toxicol. in Vitro.

[50] Chen L., Park S.M., Turner J.R., Peter M.E. (2010). Cell death in the colonic epithelium during inflammatory bowel diseases: CD95/Fas and beyond.. Inflamm. Bowel Dis..

[51] Fischbeck A., Leucht K., Frey-Wagner I., Bentz S., Pesch T., Kellermeier S., Krebs M., Fried M., Rogler G., Hausmann M., Humpf H.U. (2011). Sphingomyelin induces cathepsin D-mediated apoptosis in intestinal epithelial cells and increases inflammation in DSS colitis.. Gut.

[52] Qiu W., Wu B., Wang X., Buchanan M.E., Regueiro M.D., Hartman D.J., Schoen R.E., Yu J., Zhang L. (2011). PUMA-mediated intestinal epithelial apoptosis contributes to ulcerative colitis in humans and mice.. J. Clin. Invest..

[53] Tambuwala M.M., Cummins E.P., Lenihan C.R., Kiss J., Stauch M., Scholz C.C., Fraisl P., Lasitschka F., Mollenhauer M., Saunders S.P. (2010). Loss of prolyl hydroxylase-1 protects against colitis through reduced epithelial cell apoptosis and increased barrier function.. Gastroenterology.

[54] Gitter A.H., Bendfeldt K., Schulzke J.D., Fromm M. (2000). Leaks in the epithelial barrier caused by spontaneous and TNF-alpha-induced single-cell apoptosis.. FASEB J..

[55] Araki Y., Mukaisyo K., Sugihara H., Fujiyama Y., Hattori T. (2010). Increased apoptosis and decreased proliferation of colonic epithelium in dextran sulfate sodium-induced colitis in mice.. Oncol. Rep..

[56] Leek M.D., Griffiths G.D., Green M.A. (1989). Intestinal pathology following intramuscular ricin poisoning.. J. Pathol..

[57] Shifrin V.I., Anderson P. (1999). Trichothecene mycotoxins trigger a ribotoxic stress response that activates c-Jun *N*-terminal kinase and p38 mitogen-activated protein kinase and induces apoptosis.. J. Biol. Chem..

[58] Allan L.A., Clarke P.R. (2009). Apoptosis and autophagy: Regulation of caspase-9 by phosphorylation.. FEBS J..

[59] Moon Y., Yang H., Kim Y.B. (2007). Up-regulation of early growth response gene 1 (EGR-1) via ERK1/2 signals attenuates sulindac sulfide-mediated cytotoxicity in the human intestinal epithelial cells.. Toxicol. Appl. Pharmacol..

[60] Moon Y., Yang H., Lee S.H. (2007). Modulation of early growth response gene 1 and interleukin-8 expression by ribotoxin deoxynivalenol (vomitoxin) via ERK1/2 in human epithelial intestine 407 cells.. Biochem. Biophys. Res. Commun..

[61] Lee S.Y., Lee M.S., Cherla R.P., Tesh V.L. (2008). Shiga toxin 1 induces apoptosis through the endoplasmic reticulum stress response in human monocytic cells.. Cell Microbiol..

[62] Yang H., Park S.H., Choi H.J., Do K.H., Kim J., An T.J., Lee S.H., Moon Y. (2010). Mechanism-based alternative monitoring of endoplasmic reticulum stress by 8-keto-trichothecene mycotoxins using human intestinal epithelial cell line.. Toxicol. Lett..

[63] Park S.H., Choi H.J., Yang H., Do K.H., Kim J., Moon Y. (2010). Repression of peroxisome proliferator-activated receptor gamma by mucosal ribotoxic insult-activated CCAAT/enhancer-binding protein homologous protein.. J. Immunol..

[64] Yang H., Choi H.J., Park S.H., Kim J.S., Moon Y. (2009). Macrophage inhibitory cytokine-1 (MIC-1) and subsequent urokinase-type plasminogen activator mediate cell death responses by ribotoxic anisomycin in HCT-116 colon cancer cells.. Biochem. Pharmacol..

[65] Brown D.A., Ward R.L., Buckhaults P., Liu T., Romans K.E., Hawkins N.J., Bauskin A.R., Kinzler K.W., Vogelstein B., Breit S.N. (2003). MIC-1 serum level and genotype: Associations with progress and prognosis of colorectal carcinoma.. Clin. Cancer Res..

[66] Johnen H., Lin S., Kuffner T., Brown D.A., Tsai V.W., Bauskin A.R., Wu L., Pankhurst G., Jiang L., Junankar S. (2007). Tumor-induced anorexia and weight loss are mediated by the TGF-beta superfamily cytokine MIC-1.. Nat. Med..

[67] Nakamura T., Scorilas A., Stephan C., Yousef G.M., Kristiansen G., Jung K., Diamandis E.P. (2003). Quantitative analysis of macrophage inhibitory cytokine-1 (MIC-1) gene expression in human prostatic tissues.. Br. J. Cancer.

[68] Baek S.J., Wilson L.C., Eling T.E. (2002). Resveratrol enhances the expression of non-steroidal anti-inflammatory drug-activated gene (NAG-1) by increasing the expression of p53.. Carcinogenesis.

[69] Martinez J.M., Sali T., Okazaki R., Anna C., Hollingshead M., Hose C., Monks A., Walker N.J., Baek S.J., Eling T.E. (2006). Drug-induced expression of nonsteroidal anti-inflammatory drug-activated gene/macrophage inhibitory cytokine-1/prostate-derived factor, a putative tumor suppressor, inhibits tumor growth.. J. Pharmacol. Exp. Ther..

[70] Yang H., Park S.H., Choi H.J., Moon Y. (2010). The integrated stress response-associated signals modulates intestinal tumor cell growth by NSAID-activated gene 1 (NAG-1/MIC-1/PTGF-beta).. Carcinogenesis.

[71] Bauskin A.R., Zhang H.P., Fairlie W.D., He X.Y., Russell P.K., Moore A.G., Brown D.A., Stanley K.K., Breit S.N. (2000). The propeptide of macrophage inhibitory cytokine (MIC-1), a TGF-beta superfamily member, acts as a quality control determinant for correctly folded MIC-1.. EMBO J..

[72] Khuu C.H., Barrozo R.M., Hai T., Weinstein S.L. (2007). Activating transcription factor 3 (ATF3) represses the expression of CCL4 in murine macrophages.. Mol. Immunol..

[73] Baek S.J., Kim J.S., Moore S.M., Lee S.H., Martinez J., Eling T.E. (2005). Cyclooxygenase inhibitors induce the expression of the tumor suppressor gene EGR-1, which results in the up-regulation of NAG-1, an antitumorigenic protein.. Mol. Pharmacol..

[74] Senapati S., Rachagani S., Chaudhary K., Johansson S.L., Singh R.K., Batra S.K. (2010). Overexpression of macrophage inhibitory cytokine-1 induces metastasis of human prostate cancer cells through the FAK-RhoA signaling pathway.. Oncogene.

[75] Araki Y., Sugihara H., Hattori T. (2006). *In vitro* effects of dextran sulfate sodium on a Caco-2 cell line and plausible mechanisms for dextran sulfate sodium-induced colitis.. Oncol. Rep..

[76] Vetuschi A., Latella G., Sferra R., Caprilli R., Gaudio E. (2002). Increased proliferation and apoptosis of colonic epithelial cells in dextran sulfate sodium-induced colitis in rats.. Dig. Dis. Sci..

[77] Arai N., Mitomi H., Ohtani Y., Igarashi M., Kakita A., Okayasu I. (1999). Enhanced epithelial cell turnover associated with p53 accumulation and high p21WAF1/CIP1 expression in ulcerative colitis.. Mod. Pathol..

[78] Shinozaki M., Watanabe T., Kubota Y., Sawada T., Nagawa H., Muto T. (2000). High proliferative activity is associated with dysplasia in ulcerative colitis.. Dis Colon Rectum..

[79] Yang H., Park S.H., Choi H.J., Moon Y. (2009). Epithelial cell survival by activating transcription factor 3 (ATF3) in response to chemical ribosome-inactivating stress.. Biochem. Pharmacol..

[80] Liang G., Wolfgang C.D., Chen B.P., Chen T.H., Hai T. (1996). ATF3 gene. Genomic organization, promoter, and regulation. J. Biol. Chem..

[81] Jiang H.Y., Wek S.A., McGrath B.C., Lu D., Hai T., Harding H.P., Wang X., Ron D., Cavener D.R., Wek R.C. (2004). Activating transcription factor 3 is integral to the eukaryotic initiation factor 2 kinase stress response.. Mol. Cell Biol..

[82] Doller A., Akool el S., Huwiler A., Muller R., Radeke H.H., Pfeilschifter J., Eberhardt W. (2008). Posttranslational modification of the AU-rich element binding protein HuR by protein kinase Cdelta elicits angiotensin II-induced stabilization and nuclear export of cyclooxygenase 2 mRNA.. Mol. Cell Biol..

[83] Bush K.T., George S.K., Zhang P.L., Nigam S.K. (1999). Pretreatment with inducers of ER molecular chaperones protects epithelial cells subjected to ATP depletion.. Am. J. Physiol..

[84] Pluquet O., Qu L.K., Baltzis D., Koromilas A.E. (2005). Endoplasmic reticulum stress accelerates p53 degradation by the cooperative actions of Hdm2 and glycogen synthase kinase 3beta.. Mol. Cell Biol..

[85] Reimertz C., Kogel D., Rami A., Chittenden T., Prehn J.H. (2003). Gene expression during ER stress-induced apoptosis in neurons: Induction of the BH3-only protein Bbc3/PUMA and activation of the mitochondrial apoptosis pathway.. J. Cell Biol..

[86] Zhang F., Hamanaka R.B., Bobrovnikova-Marjon E., Gordan J.D., Dai M.S., Lu H., Simon M.C., Diehl J.A. (2006). Ribosomal stress couples the unfolded protein response to p53-dependent cell cycle arrest.. J. Biol. Chem..

[87] Yang H., Chung D.H., Kim Y.B., Choi Y.H., Moon Y. (2008). Ribotoxic mycotoxin deoxynivalenol induces G2/M cell cycle arrest via p21Cip/WAF1 mRNA stabilization in human epithelial cells.. Toxicology.

[88] el-Deiry W.S., Harper J.W., O’Connor P.M., Velculescu V.E., Canman C.E., Jackman J., Pietenpol J.A., Burrell M., Hill D.E., Wang Y. (1994). WAF1/CIP1 is induced in p53-mediated G1 arrest and apoptosis.. Cancer Res..

[89] el-Deiry W.S., Tokino T., Velculescu V.E., Levy D.B., Parsons R., Trent J.M., Lin D., Mercer W.E., Kinzler K.W., Vogelstein B. (1993). WAF1, a potential mediator of p53 tumor suppression. Cell.

[90] Jess T., Simonsen J., Nielsen N.M., Jorgensen K.T., Bager P., Ethelberg S., Frisch M. (2011). Enteric *Salmonella* or *Campylobacter* infections and the risk of inflammatory bowel disease.. Gut.

[91] Subramanian S., Campbell B.J., Rhodes J.M. (2006). Bacteria in the pathogenesis of inflammatory bowel disease.. Curr. Opin. Infect. Dis..

[92] Johannes L., Romer W. (2010). Shiga toxins-From cell biology to biomedical applications.. Nat. Rev. Microbiol..

[93] Juranic Z., Stojiljkovic M.P., Bocarov-Stancic A., Kilibarda V., Milovanovic S.R., Juranic I., Bijelogrlic S., Vuletic N., Radulovic S. (1998). T-2 toxin affects proliferation of three different neoplastic cell lines.. J. Exp. Clin. Cancer Res..

[94] Zhou X.X., Ji F., Zhao J.L., Cheng L.F., Xu C.F. (2010). Anti-cancer activity of anti-p185HER-2 ricin A chain immunotoxin on gastric cancer cells.. J. Gastroenterol. Hepatol..

[95] Lin J.Y., Tserng K.Y., Chen C.C., Lin L.T., Tung T.C. (1970). Abrin and ricin: New anti-tumour substances.. Nature.

[96] Iordanov M.S., Choi R.J., Ryabinina O.P., Dinh T.H., Bright R.K., Magun B.E. (2002). The UV (Ribotoxic) stress response of human keratinocytes involves the unexpected uncoupling of the Ras-extracellular signal-regulated kinase signaling cascade from the activated epidermal growth factor receptor.. Mol. Cell Biol..

